# Impaired Progesterone-Responsiveness of CD11c^+^ Dendritic Cells Affects the Generation of CD4^+^ Regulatory T Cells and Is Associated With Intrauterine Growth Restriction in Mice

**DOI:** 10.3389/fendo.2019.00096

**Published:** 2019-02-25

**Authors:** Kristin Thiele, Alexandra Maximiliane Hierweger, Julia Isabel Amambay Riquelme, María Emilia Solano, John P. Lydon, Petra Clara Arck

**Affiliations:** ^1^Division of Experimental Feto-Maternal Medicine, Department of Obstetrics and Fetal Medicine, University Medical Center Hamburg-Eppendorf, Hamburg, Germany; ^2^Department of Molecular and Cellular Biology, Baylor College of Medicine, Houston, TX, United States

**Keywords:** dendritic cells, IUGR, placenta, progesterone, Tregs

## Abstract

Up to 10% of pregnancies in Western societies are affected by intrauterine growth restriction (IUGR). IUGR reduces short-term neonatal survival and impairs long-term health of the children. To date, the molecular mechanisms involved in the pathogenesis of IUGR are largely unknown, but the failure to mount an adequate endocrine and immune response during pregnancy has been proposed to facilitate the occurrence of IUGR. A cross talk between the pregnancy hormone progesterone and innate immune cell subsets such as dendritic cells (DCs) is vital to ensure adequate placentation and fetal growth. However, experimental strategies to pinpoint distinct immune cell subsets interacting with progesterone *in vivo* have long been limited. In the present study, we have overcome this limitation by generating a mouse line with a specific deletion of the progesterone receptor (PR) on CD11c^+^ DCs. We took advantage of the cre/loxP system and assessed reproductive outcome in Balb/c-mated C57Bl/6 PR^flox/flox^CD11c^cre/wt^ females. Balb/c-mated C57Bl/6 PR^wt/wt^CD11c^cre/wt^ females served as controls. In all dams, fetal growth and development, placental function and maternal immune and endocrine adaptation were evaluated at different gestational time points. We observed a significantly reduced fetal weight on gestational day 13.5 and 18.5 in PR^flox/flox^CD11c^cre/wt^ females. While frequencies of uterine CD11c^+^ cells were similar in both groups, an increased frequency of co-stimulatory molecules was observed on DCs in PR^flox/flox^CD11c^cre/wt^ mice, along with reduced frequencies of CD4^+^ FoxP3^+^ and CD8^+^ CD122^+^ regulatory T (Treg) cells. Placental histomorphology revealed a skew toward increased junctional zone at the expense of the labyrinth in implantations of PR^flox/flox^CD11c^cre/wt^ females, accompanied by increased plasma progesterone concentrations. Our results support that DCs are highly responsive to progesterone, subsequently adapting to a tolerogenic phenotype. If such cross talk between progesterone and DCs is impaired, the generation of pregnancy-protective immune cells subsets such as CD4^+^ and CD8^+^ Treg cells is reduced, which is associated with poor placentation and IUGR in mice.

## Introduction

In Western societies, up to 10% of pregnancies are affected by intrauterine growth restriction (IUGR) (1), which is defined as a pathological delay in fetal growth. IUGR reduces short-term survival due to immediate neonatal complications such as asphyxia, hypothermia, hypoglycemia and immunodeficiency (2). Moreover, child development and long-term health is impaired. Thus, IUGR children have an increased risk for metabolic, immunologic and neurodevelopmental impairments (3–5). In addition, clinical studies also reveal an association between IUGR and a significantly higher incidence of metabolic, renal and cardiovascular diseases later in life of the children (6). The molecular mechanisms involved in the pathogenesis of IUGR are largely unknown. However, various pathways have been proposed to contribute to the development of IUGR including maternal, fetal, genetic, and placental factors (2).

In order to ensure adequate placentation and subsequently fetal growth, the maternal immune, and endocrine system needs to actively adapt to the semiallogenic fetus. One key features of this tailored immune response are tolerogenic dendritic cells (tDC) present at the feto-maternal interphase during early pregnancy. The tDCs are characterized by a reduced expression of co-stimulatory molecules and enhanced IL-10 production. Functionally, tDCs promote the generation of regulatory T (Treg) cells (7–9). A number of markers with immunomodulatory potential including Galectin-1 or steroid hormones have been suggested to initiate the generation of tDCs from the less stable subset of immature DCs and sustains their ability to produce IL-10 (8, 10, 11). However, due to technical limitations, it is still unknown which immunomodulatory marker directly triggers the generation of tDCs.

It was the aim of this study to overcome these limitations and address this gap in knowledge. We hereby focussed on the role of progesterone in modulating DC functions by utilizing a cell-specific knockout of the intracellular progesterone receptor (PR) on DCs, as such approach allows to investigate the direct effect of progesterone on dendritic cells and its role in pregnancy outcome.

## Materials and Methods

### Generation of PR^**flox/flox**^CD11c^**cre/wt**^ Mice

**Figure 1:**
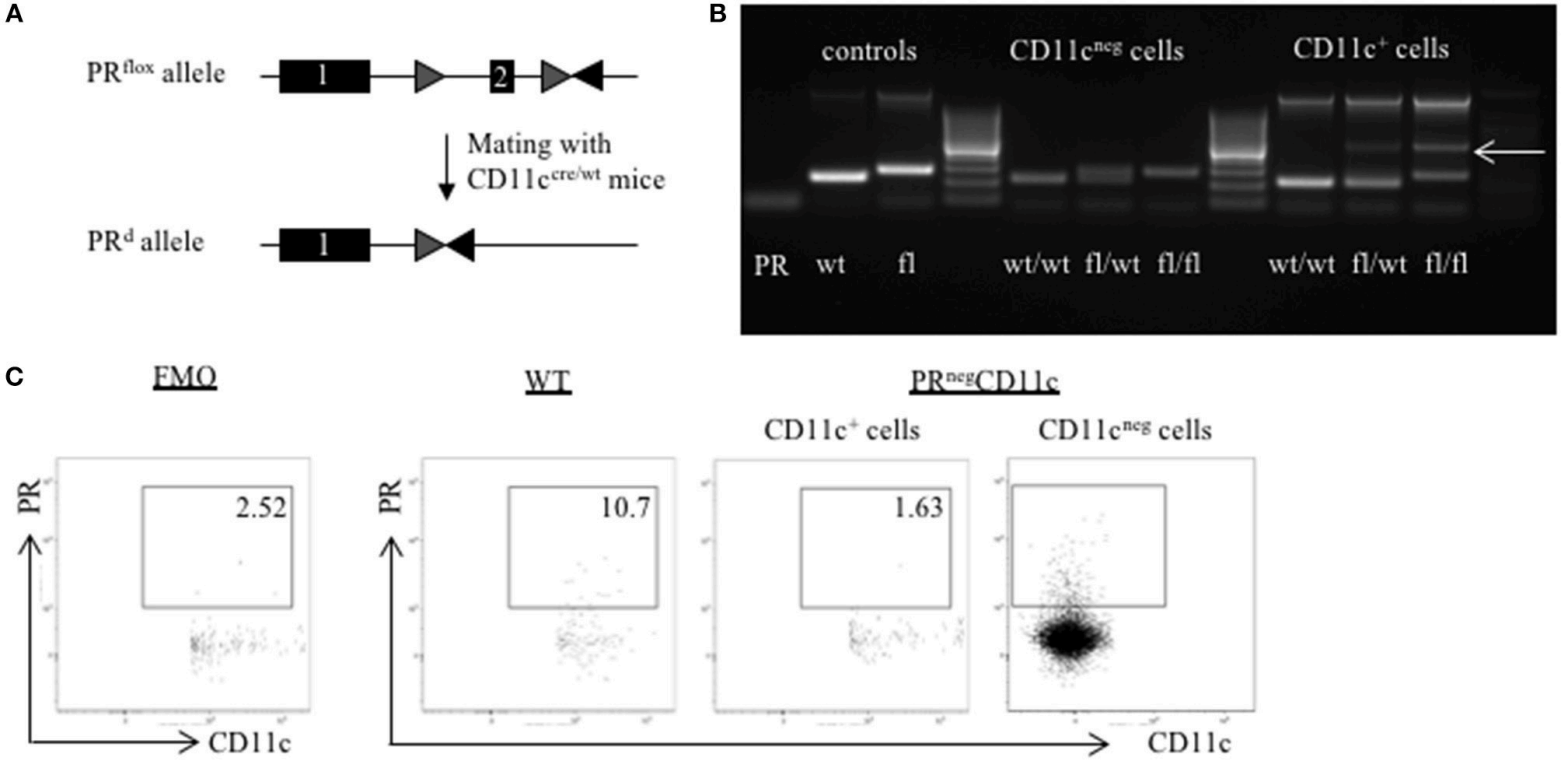
Generation of a selective knockout (KO) of the progesterone receptor (PR) on CD11c dendritic cells (DCs): **(A)** PR^flox/flox^ mice were crossed with CD11c^cre/wt^ transgenic mice resulting in recombination of the PRflox allele to a PR null allele (PRd) on CD11c^+^ cells. **(B)** The selective KO of the PR on CD11c^+^ cells was confirmed on DNA level. PCR amplicons were visualized using agarose gel electrophoresis and band sizes were 226 bp for the wildtype allele, 260 bp for the floxed allele, and 381 bp for the KO allele, respectively. **(C)** The selective KO of the PR on CD11c^+^ DCs harvested from WT and PR^neg^CD11c mice was confirmed on protein level by means of flow cytometry. The first dot plot on the left side is a fluorescence minus one (FMO) control for the PR antibody.

PR^flox/flox^ and CD11c^cre/wt^ mice were kindly provided by John P. Lydon from Baylor College of Medicine, Houston, Texas, US and Manuel Friese from University Medical Center Hamburg-Eppendorf, Hamburg, Germany, respectively. In order to generate female mice with a selective KO of the PR on CD11c dendritic cells, PR^flox/flox^ females, and CD11c^cre/wt^ males were mated in the Animal Facility of the University Medical Center Hamburg-Eppendorf (Figure 1A). Subsequently, male PR^flox/wt^ CD11c^cre/wt^ and female PR^flox/wt^ CD11c^wt/wt^ offspring were mated in order to generate PR^flox/flox^ CD11c^cre/wt^ and PR^wt/wt^ CD11c^cre/wt^ animals. Experimental mice were obtained by mating male mice with a genotype of PR^flox/flox^ CD11c^cre/wt^ and PR^wt/wt^ CD11c^cre/wt^ with PR^flox/flox^ CD11c^wt/wt^ and PR^wt/wt^ CD11c^wt/wt^ females, respectively. Hence, the cre expression was always transmitted from the father to avoid a premature potential impact of an impaired Progesterone-DC-crosstalk.

### Isolation of CD11c^+^ and CD11^neg^ Cells

In order to confirm the selective KO of the PR on CD11c dendritic cells, the spleen was harvested from WT, PR^neg^CD11c, and PR^flox/wt^ CD11c^cre/wt^ mice. Single cell suspensions were obtained as described previously (12). In brief: the tissue was mashed with the plunger of a sterile disposable syringe in circles through a 40 μm cell strainer (Falcon Cell Strainer 40 μm, BD Bioscience, VWR, Germany). The resulting cell suspension was centrifuged for 8 min with 450 × g at 4°C and the supernatant was discarded. Subsequently, a red cell blood (RBC) lysis was performed using RBC lysis buffer (eBioscience, San Diego, CA) according to the manufacturer's instruction. After centrifugation the cell pellet was resuspended in MACS-Buffer (1xPBS, 0.5% BSA. 2 mM EDTA). CD11c^+^ cells were enriched by magnetic cell separation using the CD11c MicroBeads UltraPure mouse kit (MACS®, Miltenyi Biotec, Bergisch-Gladbach, Germany) according to the manufacturer's instruction. Finally, fluorescence-activated cell sorting of CD11c^+^ and CD11^neg^ cells was performed to achieve highest purity.

### DNA Isolation and Polymerase Chain Reaction (PCR)

DNA was isolated from mouse tail and CD11c^+^ and CD11^neg^ cells obtained from WT, PR^neg^CD11c and PR^flox/wt^ CD11c^cre/wt^ mice using the DNeasy Kit (Qiagen) according to the manufacturer's protocol.

PCR analysis was performed as 3-Primer-PCR in 50 μl reactions using the Mastercycler® nexus GX2 (Eppendorf). Primer sequences used were previously published (13) and ordered from TIB Molbiol. The PCR program consisted of initial 94°C for 10 min followed by 30 cycles: 94°C for 1 min, 55°C for 1 min, and 72°C for 2 min. Amplicons were visualized using Agarose gel electrophoresis. Expected band sizes were 226bp for the wildtype allele, 260 bp for the floxed allele and 381 bp for the KO allele.

### Timed Pregnancies

Eight to ten weeks old PR^flox/flox^CD11c^cre/wt^ female mice with a C57Bl/6 background were mated to Balb/c male mice. Aged-matched Balb/c-mated PR^wt/wt^CD11c^cre/wt^ females served as controls in order to control for unwanted side effects due to the expression of the cre. For simplicity we will refer to PR^flox/flox^CD11c^cre/wt^ mice as PR^neg^CD11c and to PR^wt/wt^CD11c^cre/wt^ control mice as WT.

The presence of a vaginal plug in the morning was considered as gestation day (gd) 0.5. Maternal weight control on gd 8.5 and 10.5 was conducted to confirm pregnancy. Animals were kept under 12 h light/ dark cycles and received food and water *ad libitum*. All experiments were performed in accordance with the animal ethics approval given by the State Authority of Hamburg (ORG_763).

### Tissue Harvesting

On gd 13.5 and 18.5, mice were anesthetized by carbon dioxide ventilation, a blood sample was collected by retro bulbar puncture and subsequently mice were sacrificed by cervical dislocation. The abdomen was opened and the uterus-draining lymph node was harvested and kept in PBS on ice. The uterus was removed and stored in HBSS on ice immediately after the fetuses were isolated from the amniotic membranes to determine fetal weight. Placentas were either stored at −20°C in RNAlater (Ambion by Life Technologies GmbH) or embedded in biopsy cassettes and stored in 4% Formaldehyde solution (36.5–38%, Sigma-Aldrich, St, Louis, US) for 24 h before transfer into 1% Formaldehyde solution for long-term storage. Maternal ovaries were also preserved in RNAlater.

### Pregnancy Outcome

Number of implantations and abortions was assessed per pregnant female. The abortion rate refers to the number of fetuses resorbed per litter using the following equation: (number of abortions/number of implantations) ^*^ 100.

### Tissue Processing

Single cell suspensions of maternal lymph nodes and uteri were obtained as described before (14). In brief, maternal lymph nodes were passed through a cell strainer and after centrifugation at 450 g for 8 min at 4°C, the cell pellet was resuspended in PBS. The uterus was enzymatically digested using 200 U/mL hyaluronidase (Sigma-Aldrich), 1 mg/mL collagenase VIII type (Sigma-Aldrich), and 1 mg/mL bovine serum albumin fraction V (Sigma-Aldrich) dissolved in 5 mL HBSS. Subsequently, the uterus was incubated twice for 20 min in a 37°C water bath with agitation. Intermediately, the solution was recovered and filtered through a mesh. The solution was centrifuged at 450 × g for 10 min at 4°C and resuspended PBS.

Number of viable leukocytes in both tissues was obtained by counting the cells using a Neubauer chamber upon adding Trypan Blue stain (0.4 %, Life Technologies GmbH, Darmstadt, Germany).

### Flow Cytometry

For flow cytometric analyses, 1.0 × 10^6^ maternal lymph node and uterus cells were used. Non-specific binding was blocked by rat anti-mouse CD16/CD32 Mouse Fragment crystallizable (Fc) Block (1:200, BD Bioscience) and Normal Rat Serum (1:100, eBioscience) for 15 min at 4°C. Subsequently, the cells were incubated with the respective antibodies for 30 min for surface and intracellular stainings. Antibodies used in this study are displayed in Table 1. In case of solely surface staining, 7-amino-actinomycin D (7AAD, 1:400, Biolegend) was used to identify dead cells. For intracellular staining, cells were fixed and permeabilized using Foxp3 Fixation/Permeabilization Concentrate and Diluent (eBioscience). Dead/live staining was performed with eFluor 506 viability dye (eBioscience).

**Table 1:** Summary of antibodies used in the present study.

**Target antigen**	**Fluorochrome**	**Clone**	**Dilution**	**Source**
anti-CD45	Allophycocyanin (APC)-Cyanine (Cy)7	30-F11	1:400	BD
anti-CD3	R-phycoerythrin (PE) Cy7	145-2C11	1:200	Biolegend
anti-CD8	Brilliant Violet (BV) 650	53-6.7	1:100	Biolegend
anti-CD4	Pacific Blue	RM4-5	1:400	Biolegend
anti-FoxP3	PE	FJK-16s	1:200	eBioscience
anti-CD122	PerCP eFluor 710	TM-b1	1:100	eBioscience
F4/80	BV421	BM8	1:100	Biolegend
anti-CD11c	BV785	N418	1:100	Biolegend
MHCII	APC	M5/144.15.2	1:200	BD
anti-CD80	BV605	16-10A1	1:100	Biolegend
anti-CD86	BV605	GL-1	1:100	Biolegend

In order to quantify the expression of the PR by flow cytometry, cells were stained with the respective surface markers and subsequently permeabilized with Cytofix/Cytoperm (eBioscience) according to the manufacturer's instructions. Afterwards, cells were blocked with mouse serum preventing non-specific binding of antibodies to intracellular proteins and then incubated with Progesterone Receptor Monoclonal Antibody (R.809.9, Invitrogen) for 30 min. A FITC-conjugated secondary antibody (1:200) was added for additional 20 min.

Flow cytometric data were acquired using a BD LSRFortessa II (BD Biosciences) and analyzed using FlowJo (Tree Star, Ashland, OR, USA).

### Placenta Histology

Placentas harvested on gd 13.5 and 18.5 were embedded in paraffin. Subsequently, 4 μm thick histological sections were prepared at the mid-sagittal plane using a microtome (SM2010R, Leica, Bensheim, Germany). Slides were dewaxed and rehydrated using xylene and ethanol. Masson-Goldner trichrome staining was performed following standard protocol (15). Subsequently slides were also scanned with a Mirax Midi Slide Scanner. Histomorphological analyses of placental areas were performed by two independent observers using Pannoramic Viewer (3DHistech Kft. Budapest, Hungary).

### Progesterone Analysis

Maternal blood samples were centrifuged at 10,000 g for 20 min at 4°C and the supernatant plasma was immediately frozen at −20°C. For progesterone analysis, plasma samples were diluted 1:200 using ELISA Buffer and measured with a competitive immunoassay (Progesterone ELISA Kit, Cayman Chemical, Michigan, USA) on a NanoQuant (Tecan Group AG, Männedorf, Switzerland) according to manufacturer's instructions.

### Theiler Scoring of Fetuses on gd13.5

Fetal development of mouse embryos was evaluated by observation of Bouin-fixed fetuses under a Zeiss Stemi 2000-C stereomicroscope according to Theiler's description (16). Main criteria to differentiate the developmental stages at gd 13.5 have been the formation of the pinna, fingers and feet, and the presence or absence of 5 rows of whiskers.

### RNA Isolation and cDNA Synthesis

Following tissue harvesting, half of the placentas were preserved in RNAlater at −20°C. Tissue homogenization was carried out using micro packaging vials with ceramic beads (1.4 mm) in a Precellys® 24 Tissue Homogenizer (Peqlab). RNA isolation and DNA digestion were conducted by use of RNeasy Plus Universal Mini Kit (QIAGEN) and DNA-free Kit (Applied Biosystems by Thermo Fisher), respectively. cDNA synthesis was performed with random primers (Invitrogen by Thermo Fisher). Concentration and purity of RNA and cDNA were assessed employing NanoQuant (Tecan).

### Quantitative Real-Time Polymerase Chain Reaction (qRT-PCR)

Gene expression analyses of ovarian tissue was performed using gene expression assays (Applied Biosystems by Thermo Fisher) for steroidogenic acute regulatory protein (Star, Mm00441558_m1), 3β-hydroxysteroid dehydrogenase (Hsd3b1, Mm01261921_mH), and 20αHSD (Akr1c18, Mm00506289_m1). Beta-actin (Actb) and ubiquitin C (Ubc) served as endogenous control to normalize cDNA content. Gene expression analyses of placental tissue were carried out using gene expression assays (Applied Biosystems by Thermo Fisher) for the following targets: insulin like growth factor 1 (Igf1, Mm00439560_m1), hydroxysteroid 11-beta dehydrogenase (Hsd11b) 1 and 2 (Mm00476182_m1 and Mm01251104_m1), placental growth factor (Plgf, Mm00435613_m1), epidermal growth factor (Egf, Mm00438696_m1), vascular endothelial growth factor A (Vegf, Mm00437306_m1), B cell leukemia/lymphoma 2 (Bcl2, Mm00477631_m1) and soluble FMS-like tyrosine kinase 1 (sFlt1, Mm00438980_m1), heme oxygenase 1 (Hmox1, Mm00516005_m1), galectin-1 (Gal-1, Mm00839408_g1), and placental lactogen II (Prl3b1, Mm00435852_m1). RNA polymerase II subunit A (Polr2a, Mm00839502_m1) and ubiquitin C (Ubc, Mm02525934_g1) served as endogenous controls (17). All reactions were performed in 50 cycles using a standard two-step RT-PCR: initial 50°C for 2 min and 95°C for 10 min, 15 s denaturation at 95°C and 60 s annealing and extension at 60°C with the NanoQuant5 Real-Time PCR System (Applied Biosystems) and the corresponding software. The fold change of PR^neg^CD11c over WT control expression was calculated employing the ΔΔCt method (18).

### Statistical Analysis

Statistical analysis was performed using GraphPad Prism version 7.0 (GraphPad Software, La Jolla, CA, USA). All results are expressed as means ± standard error of the mean (SEM). Means between groups were compared using unpaired *t*-test. Welch's *t*-test was used in case of unequal standard deviation and unequal sample sizes between groups (19).

## Results

### Confirmation of the Selective Knockout of the PR on CD11c^+^ DCs

In order to confirm the selective knockout of the PR on CD11c^+^ DCs on a DNA level, we performed genotyping of tail biopsies and CD11c^+^ and CD11^neg^ cells of WT, PR^flox/wt^CD11c^cre/wt^ and PR^neg^CD11c mice, respectively. The agarose gel shows the amplicons of tail biopsies from WT mice at the expected band size of 226 bp and from PR^neg^CD11c at 260 bp. PCR of sorted CD11c^neg^ spleen cells from the respective genotypes resulted also in a 226 bp amplicon for WT and in a 260 bp amplicon for PR^neg^CD11c while cells from heterozygote mice showed both bands. Equally, PCR of sorted CD11c^+^ spleen cells from WT mice resulted in a 226 pb amplicon. CD11c^+^ spleen cells from PR^flox/wt^CD11c^cre/wt^ and PR^neg^CD11c animals exhibit a band of 381 bp for the KO allele (Figure 1B). In order to confirm this selective KO also on the protein level, we used a monoclonal antibody against the PR for detection by flow cytometry. CD11c^+^ cells harvested from PR^neg^CD11c mice showed a lower frequency of the PR on DC compared to cells isolated from WT mice (Figure 1C). A positive PR expression could be confirmed on CD11c^neg^ cells from PR^neg^CD11c mice. However, as we also detected CD11c^pos^ PR^pos^ cells at low frequencies among cells isolated from PR^neg^CD11c mice, unspecific binding of the secondary antibody must be assumed. Alternatively, the cre recombinase may not exhibit 100% efficiency.

### Impaired Progesterone-Responsiveness of CD11c^+^ DCs Affects Fetal Growth

**Figure 2:**
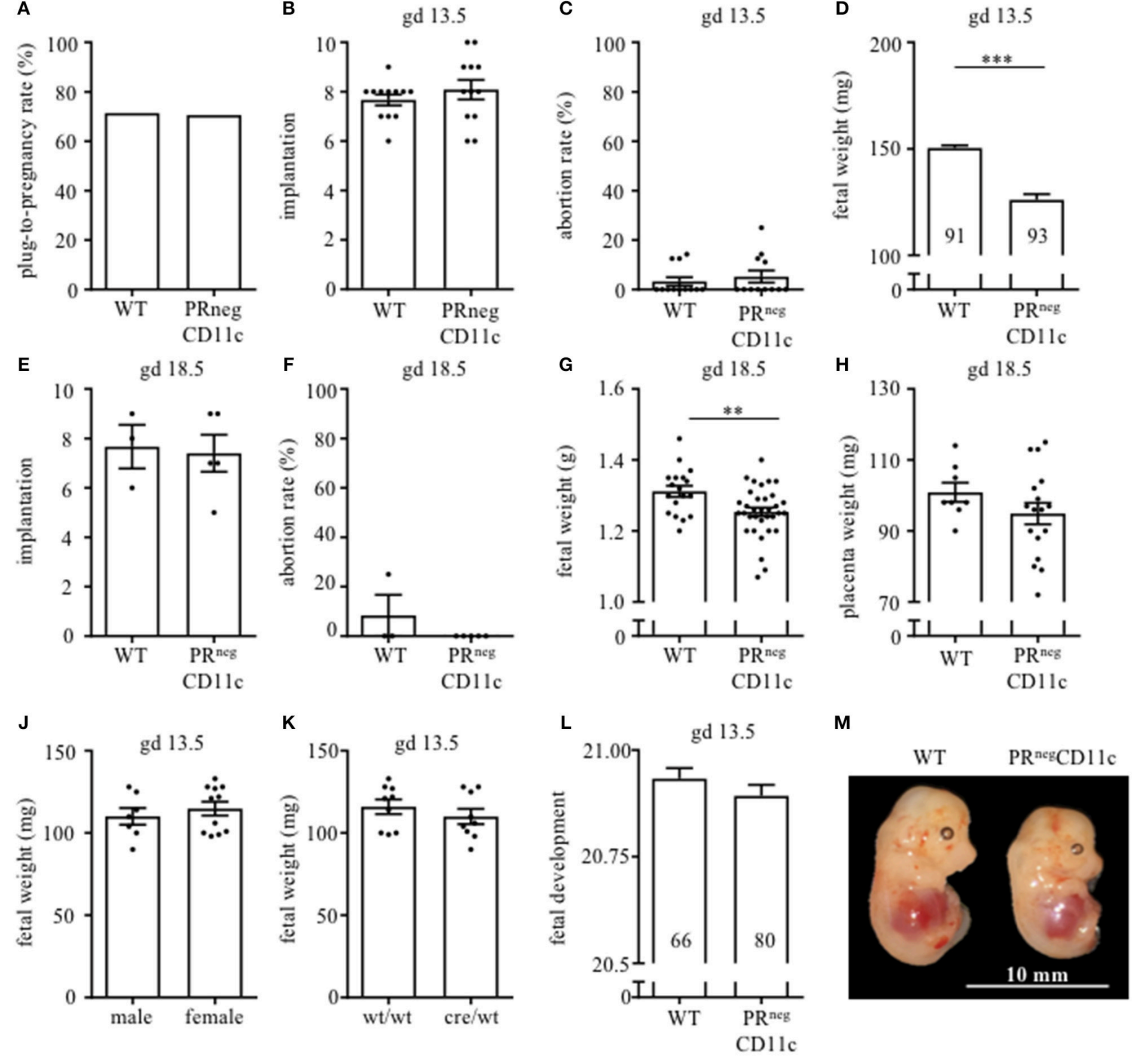
Impaired progesterone-responsiveness of CD11c^+^ dendritic cells affects fetal growth: WT and PR^neg^CD11c female mice were allogenically mated to Balb/c males and pregnancy outcome was assessed, maternal phenotype is always indicated below: **(A)** Plug-to-pregnancy rate. On gestational day (gd) 13.5, we assessed number implantation **(B)**, abortion rate **(C)** and fetal weight **(D)**. For gd 18.5, number implantation **(E)**, abortion rate **(F)**, fetal weight **(G)**, and placental weight **(H)** were determined. Fetal weight of fetuses from PR^neg^CD11c dams is further shown dependent on fetal sex **(J)** and fetal genotype **(K)**. **(L)** Fetal development was scored on gd13.5 according to Theiler criteria and a representative picture from gd 13.5 fetuses of WT (left) and PR^neg^CD11c (right) mice is shown in **(M)**, white line in the picture denote 10 mm. Data are represented as mean ± SEM. ***p ≤ 0.001, **p ≤ 0.01.

Pregnancy rate did not significantly differ between WT and PRnegCD11c female mice (Figure 2A). Pregnancy outcome was assessed on gd 13.5 as well as 18.5. We could not identify any modification with regards to number of implantations (Figures 2B,E) and abortion rate (Figures 2C,F) in litters from WT and PR^neg^CD11c females. However, we observed a significantly reduced fetal weight on gestational day 13.5 and 18.5 in litters of PR^neg^CD11c females (Figures 2D,G) along with a minor tendency toward a decreased placental weight on gd 18.5 (Figure 2H). We did not observe sex-specific effects, as a similar fetal weight could be observed in male and female offspring (Figure 2J). Further, the chosen mating combination yields to two fetal genotypes: PR^flox/wt^CD11c^cre/wt^ and PR^flox/wt^CD11c^wt/wt^. In order to exclude an impact of the fetal expression of the cre recombinase on the fetal weight, we assessed the fetal genotype from 2 litters and could exclude that the fetal genotype caused alterations of the fetal weight (Figure 2K). Using Theiler staging as a criterion to assess fetal development, we observed only a minor delay in fetuses from PR^neg^CD11c mother (Figure 2L). A representative photographic image of fetuses from WT and PR^neg^CD11c mice in shown in Figure 2M.

### Impaired Progesterone-Responsiveness of CD11c^+^ DCs Affects Maternal Immune Adaptation

**Figure 3:**
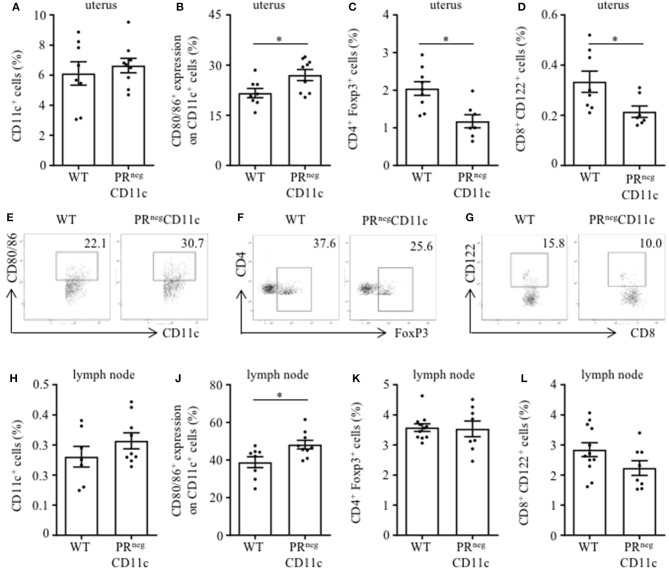
Impaired progesterone-responsiveness of CD11c^+^ dendritic cells (DCs) affects maternal immune adaptation: WT and PR^neg^CD11c female mice were allogenically mated and flow cytometric analysis was performed on gestation day 13.5. Graphs present the frequencies of **(A)** CD11c^+^ DCs, **(B)** the co-expression of CD80 and CD86, **(C)** CD4^+^FoxP3^+^ Treg cells, and **(D)** CD8^+^CD122^+^ T cells in uteri harvested from WT and PR^neg^CD11c dams. Representative dot plots display CD80/86 expression on CD11c^+^ cells **(E)**, FoxP3^+^ expression on CD4^+^ cells **(F)**, and CD122^+^ expression in CD8^+^ T cells **(G)** of WT and PR^neg^CD11c mice. Respective cell frequencies in the right corner are expressed as percentage of CD11c^+^, CD4^+^ and CD8^+^ cells, respectively. **(H–L)** Flow cytometric analysis of CD11c^+^ DCs **(H)**, the co-expression of CD80 and CD86 **(J)**, CD4^+^FoxP3^+^ Treg cells **(K)**, and CD8^+^CD122^+^ T cells **(L)** in uterus-draining lymph node harvested from WT and PR^neg^CD11c dams. Bars represent mean ± SEM. *p ≤ 0.05, unless otherwise stated, cell frequencies are expressed as percentage of living CD45^+^ cells.

Flow cytometry analysis from the uterus of gd 13.5 revealed similar frequencies of uterine CD11c^+^ cells in both groups (Figure 3A). While the co-expression of MHCII was not different between groups (data not shown), we observed increased frequencies of DCs expression co-stimulatory molecules CD80 or CD86 in PR^neg^CD11c mice (Figure 3B). Further, we identified reduced frequencies of CD4^+^ FoxP3^+^ and CD8^+^ CD122^+^ regulatory T (Treg) cells in uteri of PR^neg^CD11c dams (Figures 3C,D). Representative dot plots are shown in Figures 3E–G. We made similar observations of unaltered CD11c frequencies and increased co-expression of CD80 and CD86 in cells isolated from uterus-draining lymph nodes (Figures 3H,J), whereas no significant differences were detectable for CD4^+^ FoxP3^+^ Treg cell frequencies (Figure 3K) and CD8^+^ CD122^+^ Treg cells (Figure 3L) between WT and PR^neg^CD11c dams.

### Placental Histomorphology and Plasma Progesterone Levels Was Modulated on gd 13.5

**Figure 4:**
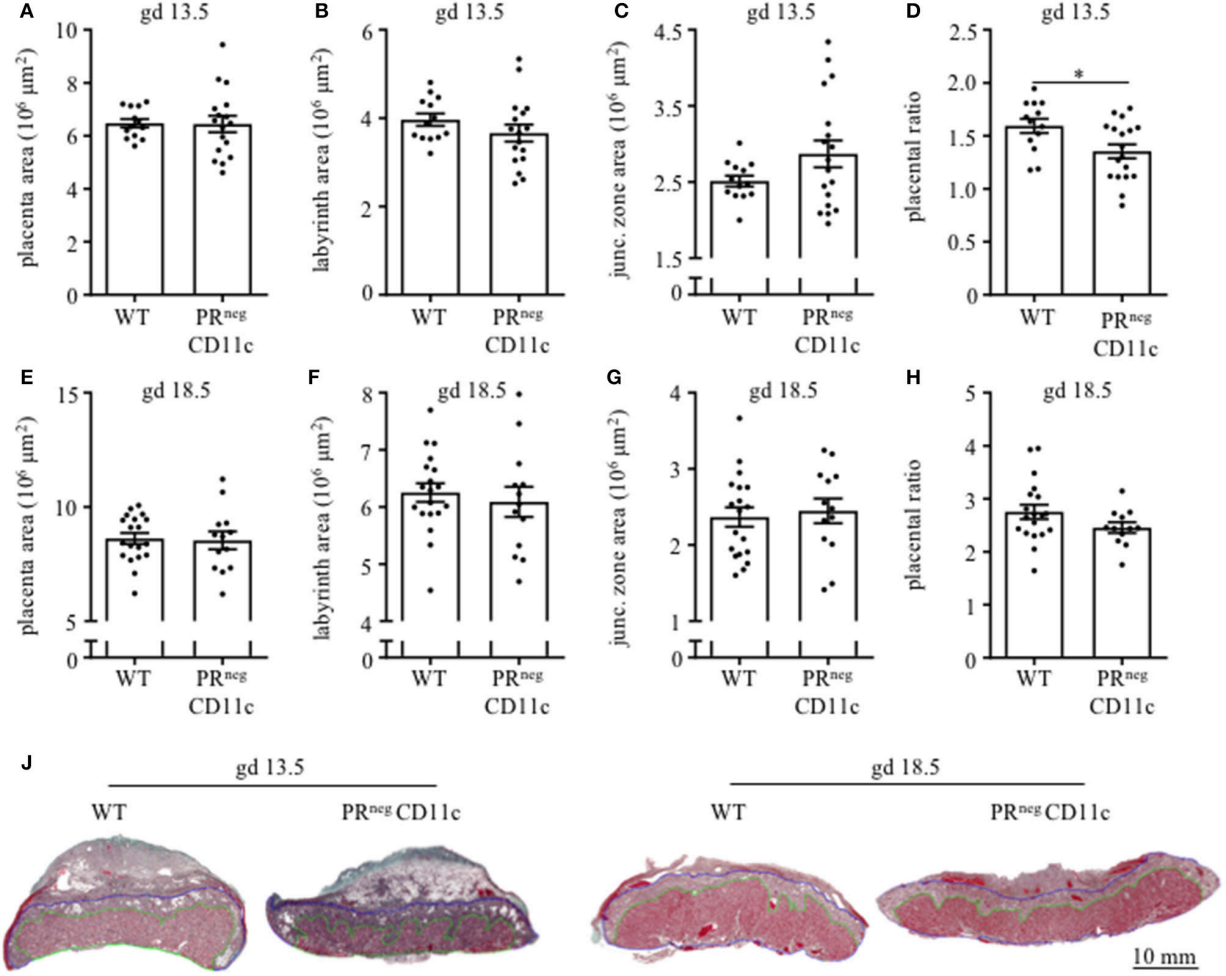
Impaired progesterone-responsiveness of CD11c^+^ dendritic cells affects placental morphology: WT and PR^neg^CD11c female mice were allogenically mated and placentas harvested on gestation day (gd) 13.5 und 18.5 were evaluated by Masson-Goldner trichrome staining allowing the differentiation of the labyrinth and junctional zone. **(A,E)** show total placenta area on gd 13.5 and 18.5, respectively. Area of the labyrinth **(B,F)** and the junctional zone **(C,G)** have been assessed and the placental ratio **(D,H)** was calculated. **(J)** Representative photomicrographs illustrating mid-sagittal sections of gd 13.5 and 18.5 placental tissue from WT (left) and PR^neg^CD11c (right) mothers, black line in the picture denotes 10 mm, green lines encircle the labyrinth, blue lines surround the junctional zone. Data are represented as mean ± SEM. *p ≤ 0.05.

Placenta morphology was assessed on gd 13.5 and 18.5 by Masson-Goldner trichrome staining on mid-sagittal sections. The overall placental surface area did not differ between groups (Figures 4A,E). However, a skew toward an increased junctional zone at the expense of the labyrinth could be detected in PR^neg^CD11c females compared to WT females on gd 13.5 (Figures 4B,C), which resulted in a significantly decreased placental ratio (labyrinth/junctional zone, Figure 4D), a proxy for placental function (20). The same observation could be made when analyzing the placentas from gd 18.5, but it did not reach statistical significance (Figures 4F–H). Representative photomicrographs from gd 13.5 and 18.5 placentas are shown in Figure 4J.

**Figure 5:**
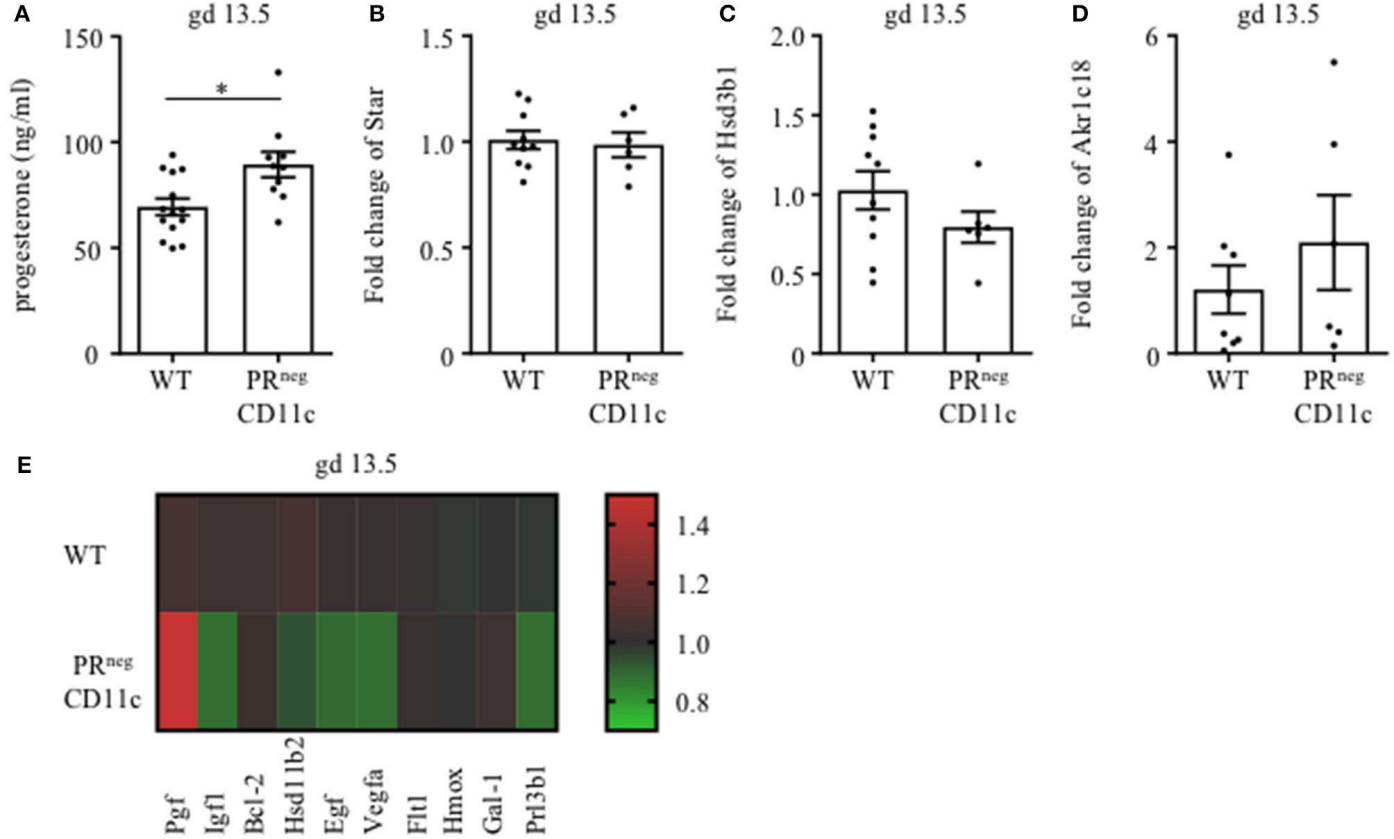
Selective knockout of the progesterone receptor (PR) on CD11c^+^ dendritic cells affects gene expression analysis of ovarian and placental tissue: **(A)** Plasma progesterone levels of WT and PR^neg^CD11c female mice on gd 13.5 as analyzed by ELISA. **(B–D)** Ovarian expression of steroidogenic acute regulatory protein (Star), 3β-hydroxysteroid dehydrogenase (Hsd3b1), and 20αHSD (Akr1c18) was assessed by qPCR from tissue harvested on gd 13.5. The fold change over WT control expression was calculated using beta-actin (Actb) and ubiquitin C (Ubc) as endogenous control and employing the ΔΔCt method. **(E)** Heatmap summarizing the placental expression of placental growth factor (Plgf), insulin like growth factor 1 *(*Igf1), B cell leukemia/lymphoma 2 (Bcl2), hydroxysteroid 11-beta dehydrogenase (Hsd11b2), epidermal growth factor (Egf), vascular endothelial growth factor A (Vegfa), soluble FMS-like tyrosine kinase 1 (sFlt1), heme oxygenase 1 (Hmox1), Galectin-1 (Gal-1), and placental lactogen II (Prl3b1) calculated by qPCR from gd 13.5 placentas. The fold change over WT control expression was calculated using RNA polymerase II subunit A (Polr2a) and Ubc as endogenous control and employing the ΔΔCt method. Bars represent mean ± SEM. *p ≤ 0.05.

We also determined plasma progesterone concentrations and observed a significant increase in PR^neg^CD11c mice compared to WT controls on gd 13.5 (Figure 5A). Subsequent qPCR analysis of maternal ovaries on gd 13.5 revealed no differences in gene expression of the ovarian steroidogenic acute regulatory protein (Star) (Figure 5B) and 3β-hydroxysteroid dehydrogenase (Hsd3b1), which converts pregnenolone to progesterone (Figure 5C). Progesterone metabolism was also not modulated in the KO line, suggested by the low, unaltered ovarian expression of 20α-hydroxysteroid dehydrogenase (Akr1c18) (Figure 5D).

We also analyzed the differential expression of placental genes that has been linked to the pathogenesis of IUGR (Figure 5E) and could demonstrate that placental growth factor (Plgf) was significantly increased in placentas from PR^neg^CD11c mothers. In contrast, significantly decreased placental expression was observed for epidermal growth factor (Egf), vascular endothelial growth factor A (Vegfa) and insulin like growth factor 1 (Igf1). In addition, hydroxysteroid 11-beta dehydrogenase 2 (Hsd11b2) and placental lactogen II (Prl3b1) showed a trend toward reduced expression in PR^neg^CD11c placentas, but did not reach levels of significance. B cell leukemia/lymphoma 2 (Bcl-2), soluble FMS-like tyrosine kinase 1 (sFlt1), heme oxygenase 1 (Hmox), and galectin-1 (Gal-1) were not affected by the selective KO of the PR.

## Discussion

The steroid hormone progesterone is known to be indispensable for successful reproduction, as demonstrated in mice carrying a null mutation of the PR gene (PR-KO mice) (21). Here, male mice do not show any abnormalities, but female mice display significant functional defects in all reproductive tissues, including the inability to ovulate, uterine hyperplasia and inflammation and severely limited mammary gland development (21).

**Figure 6:**
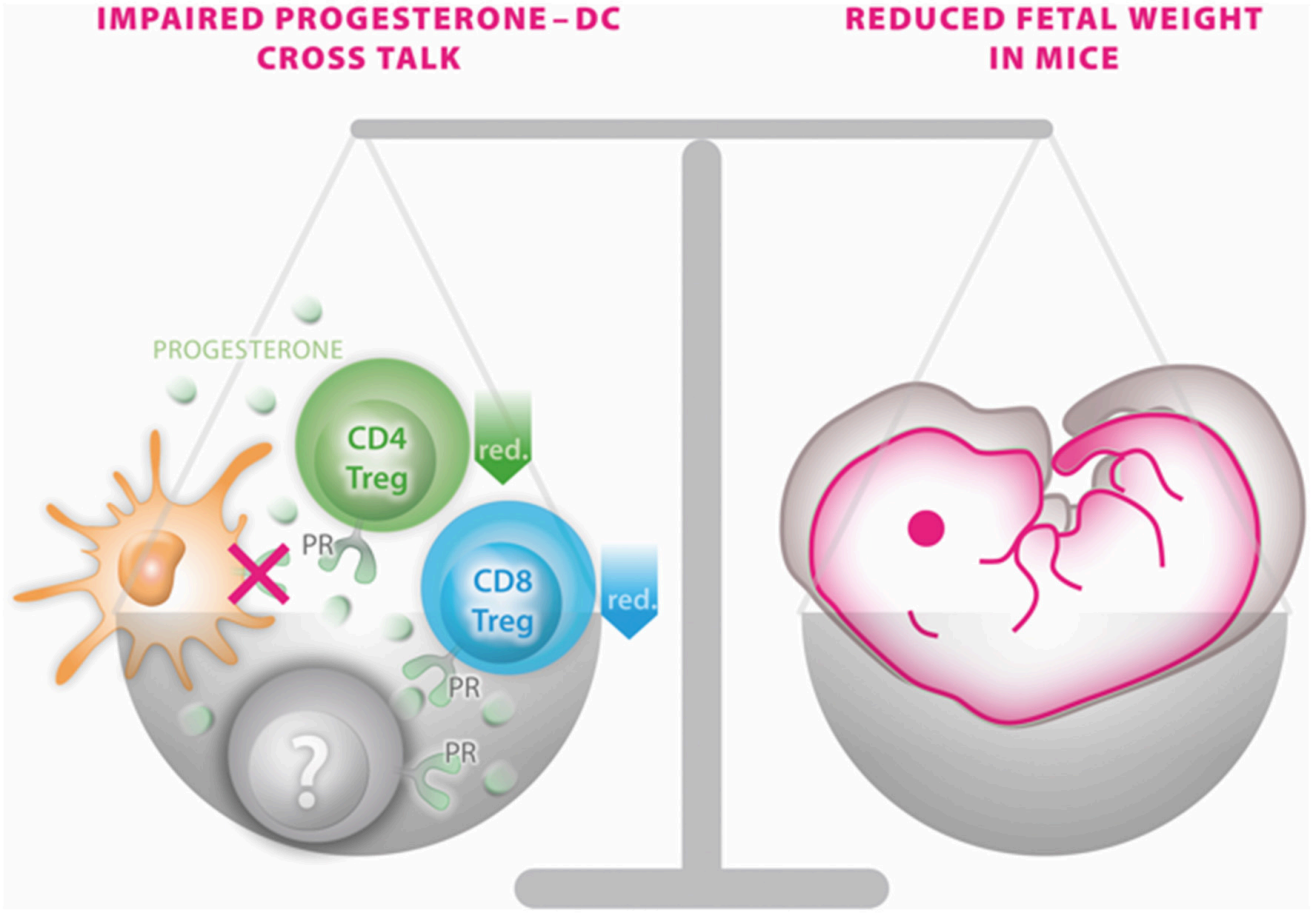
Graphical scenario depicting the impact of the selective knockout of the progesterone receptor on CD11c^+^ dendritic cells (DCs) and its consequences for fetal growth. An impaired progesterone-DC cross talk disturbs the fine-tuned balance of a functional immune response of DCs to progesterone by affecting CD4 and CD8 Treg populations (and potentially also on yet to be identified immune cells marked in gray) on one site and fetal outcome on the other side.

Due to the complexity of endocrine-immune cross talk particularly in the context of steroid actions, the generation of a mouse model enabling conditional excision of PR function (PRflox mice) in a cell- or tissue-specific manner provides a useful tool to study progesterone-dependent pathways and processes (13). Using such approach in the present study, we observed that mice devoid of PR on DCs do not exhibit major reproductive abnormalities, such as an altered plug-to-pregnancy rate or increased fetal loss rate. However, the offspring of such PR^neg^CD11c dams were severely affected by IUGR, as fetal weight was ~15% lower compared to WT dams on gd 13.5. This result was not compromised by possible alterations of the viable litter size, by fetal sex or fetal genotype as such effects were not detectable between the groups and could therefore be excluded as potential confounder. A graphical summary of the main finding is presented in Figure 6.

Our data further provide strong evidence that progesterone is directly involved in arresting DCs in a tolerogenic phenotype, mirrored by the increased expression of the co-stimulatory molecules CD80/CD86 in mice where the PR is lacking on DCs. This sheds new light on the recent recognition that progesterone promotes tolerance of the adaptive immune system during gestation via glucocorticoid receptor-dependent pathways (22) and suggests that progesterone modulates innate immune response during gestation through the PR by inducing a tolerogenic phenotype in DCs. However, the extrapolation of cellular functions solely based on the expression of cell surface markers is limited. Thus, the presumed tolerogenic function of DCs during pregnancy—or lack thereof in the absence of the PR—requires confirmation upon functional in-depth characterization in future experiments.

Such tDC may then account for the generation of CD4^+^ Treg cells, which has been suggested by published data (7–9, 23, 24). Our data support that progesterone considerably induces the generation of CD4^+^ Treg cells via DC-dependent pathways rather than direct effects on CD4^+^ T cells. However, in order to prove this causality, it will be necessary to perform adoptive transfer experiments demonstrating that replenishing Tregs in mice devoid of the PR on CD11c cells will prevent the development of the observed IUGR. Similarly, we provide evidence that the generation of a newly identified CD8^+^ CD122^+^ Treg cell subset involved in promoting fetal growth (15) is also critically dependent on the possibility of DCs to respond to progesterone. It has been suggested that progesterone upregulates placental Hmox1 expression, hereby promoting the generation of CD8^+^ CD122^+^ T cells. However, since placental expression of Hmox1 was not altered in mice lacking the PR on DCs, we propose that placental Hmox1 as well as tDCs may independently promote the generation of CD8^+^ CD122^+^ T cells.

As CD11c cells are involved in a variety of functions, e.g., thymic T cell development, one might suspect that the missing PR on CD11c cells may have an impact on the adaptive immune response even outside the context of pregnancy, but progesterone levels are rather low in pre-adolescent female mice and also during the menstrual cycle. Further, reduced CD4^+^ Treg frequencies were exclusively observed locally in the uterus of PRnegCD11c mice, whereas CD4^+^ Treg frequencies in secondary lymphoid organs or peripherally were unaffected. Hence, an effect of the missing PR on CD11c cells on the adaptive immune system in a non-pregnant state can largely be excluded.

We could pinpoint the cause for IUGR to the lack of the PR on DCs in the mother rather than the fetal genotype, as modulation was not dependent on the fetal genotype and only affected by the maternal PR expression on DCs. Modulation of fetal weight loss can result from placental insufficiency, a known contributor to IUGR (25). We could confirm such effect, as poor placental development was present in litters of PR^neg^CD11c dams. Whilst placental size and weight was unaffected by the maternal genotype, the junctional zone was increased at the expense of the labyrinth at implantation sites of PR^neg^CD11c dams. Such skew of placental functional areas might impair fetal supply with nutrients and oxygen (26).

Normally, impaired fetal growth and poor placentation is accompanied by reduced progesterone levels, as seen in response to prenatal challenges (14, 15). Hence, we were rather intrigued by the high progesterone levels in PR^neg^CD11c dams. This overproduction of progesterone, which appears to be an attempt to compensate for its failure to interact with DC, may lead to other placental modifications contributing to the development of IUGR, such as placental vasculogenesis and angiogenesis. Both are modulated by endothelium-specific molecules such as PlGF and VEGF (27, 28) which compete for the same cell surface tyrosine kinase receptors, VEGFR-1/Flt-1, but differentially induce angiogenesis; VEGF controls branching of blood vessels, while PlGF promotes a low resistance vascular network during mid- to late pregnancy (28). However, both factors are sensitive for changes in the microenvironment. As progesterone release is reduced in hypoxic condition (29), it could be speculated that high progesterone favors a hyperoxic environment. In this case, placental PlGF is increased while VEGF is decreased (30), similar to the gene expression we observed in our study. Consequently, superfluous progesterone could result in the decreased vascular branching, along with an increase in fetoplacental flow impedance via differential modulation of PlGF and VEGF (31). Moreover, the decrease of VEGF we observed in dams lacking the PR on DCs is even more striking as the competition for the same receptor should normally increase the quantity of VEGF then acting via the VEGFR-2 which is a major mediator of angiogenic responses (32). On the other hand, there are many studies postulating a decrease of PlGF in IUGR placenta (33–35). Hence, one might speculate that Plgf expression is not dependent on a functional PR-DC crosstalk and the observed over-expression is initiated to support placentation and angiogenesis. An altered placental gene expression in IUGR was reported for EGF (36), which we could also observe in placentas from PR^neg^CD11c females. Hence, EGF may be a target that requires a communication between progesterone and DCs and a lack of this communication impairs placental vascularization and consequently fetal nourishment. Additionally, IGF1 is known to facilitate the transport of glucose and amino acids across the placenta to the fetus and placental expression is reduced in IUGR and SGA fetuses associated with DNA methylation alterations (37). Our results support that the reduced placental Igf-1 expression observed in PR^neg^CD11c dams contribute to development of IUGR (38, 39). Taken together, these qPCR results pinpoint the need to characterize the mechanisms underlying placental vascularization in PR^neg^CD11c mice in future studies.

One limitation of the mouse model we here describe is that we chose CD11c-cre mice to target gene expression in DC (40). Although CD11c is widely accepted as a pan marker for conventional DCs (cDCs), CD11c is also expressed on macrophages and monocytes (41), plasmacytoid DCs and marginally on some lymphocyte subsets (42). Therefore, the CD11c cell specific deletion of the PR we here describe is not fully DC specific. A better option may be the recently generated cDC-restricted cre mouse (zDCcre) using a zinc finger transcription factor, zDC (Zbtb46, Btbd4), which is not expressed by monocytes, pDCs, or other immune cell populations (43, 44). These zDCcre mice could be used in future investigation to knock out the PR in cDCs in order to verify and compare to the present results. Nevertheless, as the large majority of CD11c^+^ cells are cDCs, our results provide strong evidence of a critical crosstalk between progesterone and DCs to ensure reproductive success.

Further, the cre-lox system itself shows certain limitations, such as the efficiency of target gene deletion, cre-mediated toxicity and undesired deletions (45). In order to avoid the two latter confounding factors, we used PR^wt/wt^CD11c^cre/wt^ mice as controls. Even if the CD11c-cre exhibits unwanted side effects, we can exclude an impact on our result, as both groups share the same potential cre-mediated toxicity burden. Upon detection of the PR gene on DNA and protein level, we observe an incomplete gene deletion on DCs. This may result from cell contamination during MACS and sorting, unspecific binding of the secondary antibody, but also be due to a minor deficiency of PR gene target deletion on CD11c^+^ cells. Although PR deletion might be incomplete, this mouse model has proven to be a suitable tool to investigate progesterone-dependent pathways in DCs.

In summary, we utilized the Cre-Lox recombination to successfully generate a DC-specific knockout of the PR in mice. We could demonstrate that a progesterone-responsiveness of dendritic cells is critical for fetal growth and facilitates the generation of pregnancy-protective CD4^+^ and CD8^+^ Tregs and adequate placentation. An association between diseases of insufficient placentation and the maturity of DCs have also been demonstrated in human placenta (46). In addition, human pregnancies complicated by IUGR were shown to exhibit peripheral blood DCs with an altered state of activation (47). These findings highlight the importance that an in-depth understanding of modulators that yield to such DC alterations must be gained, as addressed in the present manuscript.

## Author Contributions

KT and PA: conceptualization; KT, AH, JL, and MS: methodology; KT, AH, and JR: Investigation; KT, JL, and PA: resources; KT and PA: writing—original draft; All authors: comments on manuscript.

### Conflict of Interest Statement

The authors declare that the research was conducted in the absence of any commercial or financial relationships that could be construed as a potential conflict of interest.
